# Access Isn’t Enough: Evaluating the Quality of a Hospital Medical Assistance in Dying Program

**DOI:** 10.1007/s10730-022-09486-8

**Published:** 2022-08-26

**Authors:** Andrea Frolic, Marilyn Swinton, Allyson Oliphant, Leslie Murray, Paul Miller

**Affiliations:** 1 Department of Health Research Methods, Evidence and Impact, McMaster University, Hamilton, ON Canada; 2 Mohawk College, Hamilton, ON Canada; 3 Medical Assistance in Dying Program, Hamilton Health Sciences, Hamilton, ON Canada; 4 Divisions of Emergency Medicine and Palliative Care, McMaster University, Hamilton, ON Canada; 5 Program for Ethics and Ecologies of Care (PEaCE), McMaster University Medical Center, 1F9-1200 Main Street West, L8N 3Z5 Hamilton, ON Canada; 6 Department of Family Medicine, McMaster University, Hamilton, ON Canada

**Keywords:** MAiD, Medical assistance in Dying, Physician assisted dying, Hospitals, Evaluation, Qualitative, Methods, Quality Assurance, Program Evaluation, Organizational Ethics

## Abstract

Following an initial study of the needs of healthcare providers (HCP) regarding the introduction of Medical Assistance in Dying (MAiD), and the subsequent development of an assisted dying program, this study sought to determine the efficacy and impact of MAiD services following the first two years of implementation. The first of three aims of this research was to understand if the needs, concerns and hopes of stakeholders related to patient requests for MAiD were addressed appropriately. Assessing how HCPs and families perceived the quality of MAiD services, and determining if the program successfully accommodated the diverse needs and perspectives of HCPs, rounded out this quality evaluation. This research implemented a mixed-methods design incorporative of an online survey with Likert scale and open-ended questions, as well as focus groups and interviews with staff and physicians, and interviews with MAiD-involved family members. There were 356 online surveys, as well as 39 participants in six focus groups with HCP, as well as fourteen interviews with MAiD-involved family members. Participants indicated that high-quality MAiD care could only be provided with enabling resources such as policies and guidelines to ensure safe, evidence-based, standardized care, as well as a specialized, trained MAiD team. Both focus group and survey data from HCPs suggest the infrastructure developed by the hospital was effective in delivering high-quality MAiD care that supports the diverse needs of various stakeholders. This study may serve as a model for evaluating the impact and quality of services when novel and ethically-contentious clinical practices are introduced to healthcare organizations.

## Introduction

There is limited knowledge about assessing the quality of care related to Medical Assistance in Dying (MAID). Quality of MAiD care must take into account many domains (Oczkowski et al., 2020) which may be influenced by local conditions and stakeholder needs. Most of the literature on assisted dying is from jurisdictions where MAiD is provided mostly at home; however, in Ontario, most MAiD deaths since 2016 have occurred in hospitals, or through hospital-based MAiD programs (Government of Canada, 2020). Hospitals present unique challenges to MAiD services, including: interprofessional, team-based care, which often results in many providers interacting with patients/families on various aspects of their care; limited patient/family privacy; the necessity for care coordination across multiple teams; the often transient nature of therapeutic relationships; and the acuity of patient illness. Nothing has been reported in the literature regarding the success factors that support high-quality MAiD care in the specific context of a hospital setting, considering the perspectives of multiple stakeholders (MAiD providers, other health care professionals [HCPs], families and hospital leaders).

In this special issue describing the practical ethics involved in envisioning and implementing a hospital-based MAiD service, we report on a program evaluation study undertaken to capture the experiences and perceptions of a hospital-based MAiD program. The program was launched in 2016 based on an organizational ethics engagement process (Frolic & Miller, 2022) and a stakeholder needs assessment study (Frolic et al., 2022b). The evaluation study was undertaken in 2018, two years after the creation of the hospital MAiD program (Frolic et al., 2022a).

We explore changes in stakeholder attitudes over the first two years of MAiD and the integration of core values into practice. We describe quality improvement opportunities and articulate concrete ways other healthcare organizations could support a values-based approach to MAiD. This paper highlights the importance of designing clinical services for controversial practices like MAiD that strike a balance between honoring the moral diversity of our workforce, while also supporting patient access to this legal option of care.

Understanding how stakeholders in the hospital system view quality when it comes to MAiD services is critical to the successful integration of MAiD into the culture of an organization. We provide a model for other MAiD services to engage in a systematic evaluation process in order to assess the effectiveness and sustainability of a program, and its alignment with core values important to HCPs, families and hospital leaders. A commitment to rigorous program evaluation is critical to expanding our understanding of the practical ethics of providing assisted dying, and to ensuring services meet the needs of all stakeholders using an evidence-based approach.

## Background

### Literature Review

No other study of MAiD in Canada has focused on hospital-based practice. In addition, this study is unique in that it is a program evaluation that captures a 360 degree view of MAiD practice in a hospital setting. The study compares and contrasts the perspectives on hospital MAiD services from families, MAiD providers, healthcare providers and senior leaders. It goes beyond mere “satisfaction surveys” to assess the effectiveness of the MAiD infrastructure in meeting the needs of diverse groups, and its adherence to the core values and ethical practice standards of the program. It also identifies quality gaps and practical improvement opportunities. Emerging studies evaluate the perspectives of MAiD families and are frequently conducted and led by physicians and healthcare providers who are directly involved in MAiD care (Hales et al., 2019; Goldberg et al., 2019; Holmes et al., 2018). However, the family perspective presented in this manuscript constitutes a unique contribution through the use of non-clinical staff who interviewed families in non-clinical settings to better explore their lived experience, emotions and the legacy of MAiD.

### Setting and Foundational Research

Approximately 2000 patients die every year at Hamilton Health Sciences (HHS), which is the largest tertiary care center for the Hamilton-Niagara region of Ontario. It has a strong emphasis on teaching and research through a longstanding affiliation with McMaster University. Large regional programs in oncology, neurology, cardiology, geriatrics, and acute subspecialty-medicine programs are supported by the largest inpatient palliative care unit in Canada.

**Fig. 1:**
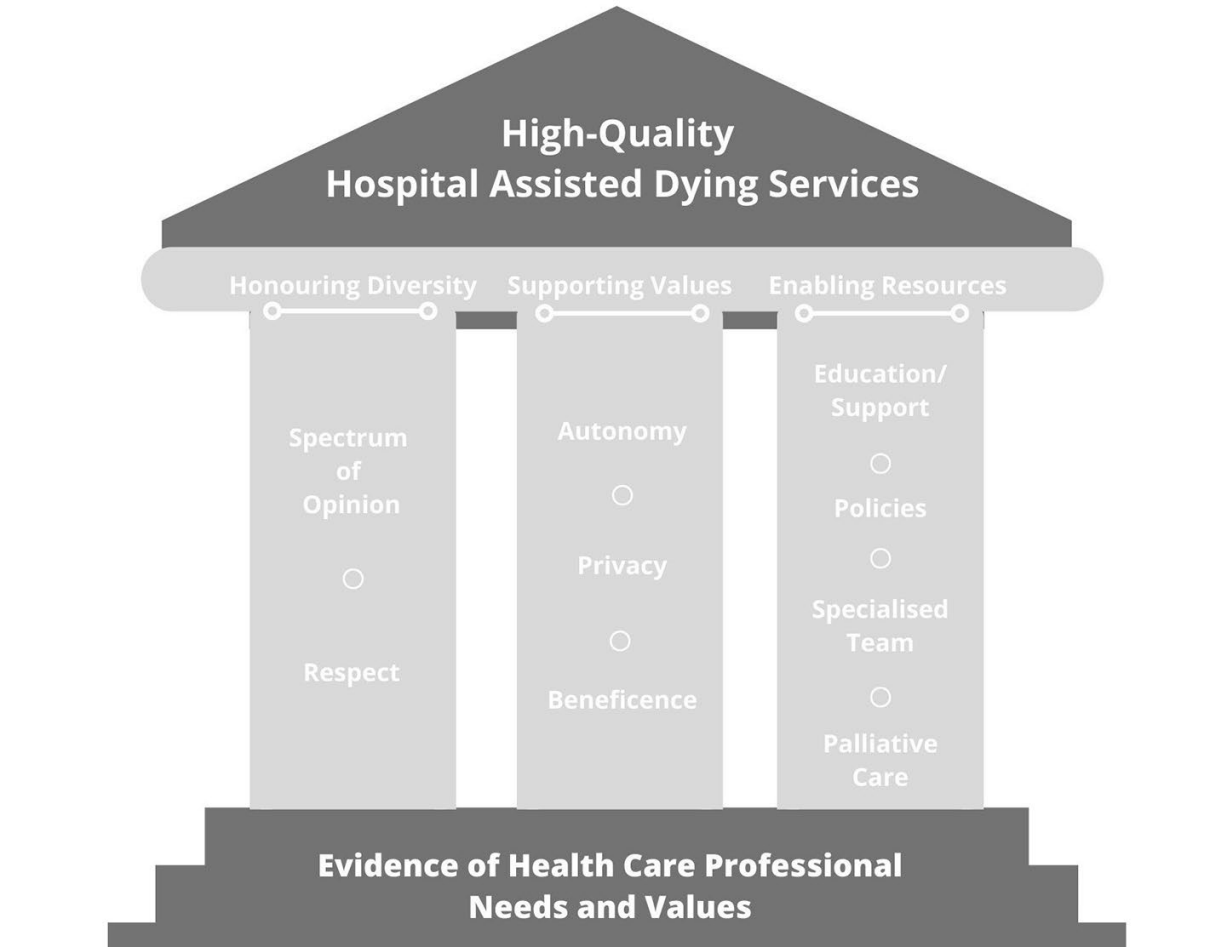
Designing Hospital MAiD Services

Development of the MAiD program at HHS was informed by the findings of a HCP stakeholder needs assessment project and an organizational ethics engagement process conducted at the organization in 2015-16, after the Supreme Court’s decision to decriminalize MAiD, but before the introduction of the legislation governing the practice of MAiD in June 2016 (Frolic et al. 2022b). Main findings of the needs assessment study are represented in Fig. 1.

Based on the results of this stakeholder needs assessment, HHS developed resources, infrastructure, protocols and tools to support and standardize MAiD practice. The key innovation of the program is the Assisted Dying Resource and Assessment Service (ADRAS), an interprofessional volunteer team comprised of about 20 health professionals with a range of expertise, including: medicine, nursing, social work, ethics, spiritual care, quality, speech language pathology, and psychology (Frolic et al., 2022a). The mandate of the ADRAS team is summarized in Table 1.0. This model of care was honored with a Palliative Care Innovation Award from the Canadian Foundation for Healthcare Improvement in 2017/18, which funded this program evaluation study.

**Table 1:** Assisted Dying Resource and Assessment Service (ADRAS) Team Charter

**Purpose**: The purpose of ADRAS is to provide a centralized consultation and referral service to responsibly and compassionately manage patient inquiries and requests for assisted dying, and to support clinical teams caring for these patients.
**Mandate**: To provide expert and effective consultation services using an interprofessional team model in alignment with legislative/regulatory requirements, professional practice guidelines and emerging best practices, and in collaboration with community partners. ADRAS provides service to HHS inpatients and outpatients, following and supporting the patient, family and healthcare team from first inquiry to the provision of assisted dying, tailoring its services to the specific needs of each situation.
**Roles and Responsibilities**:
• Develop policies and resources that ensure consistency and transparency of MAiD services, and comply with all professional and legal requirements (i.e. documentation standards and forms). • Provide standardized, high-quality MAiD assessments and provisions. • Utilize an interprofessional team structure to enable whole-person, compassionate care for patients and families and their providers. • Build capacity for responding to patient MAiD requests amongst clinical teams through coaching, education and collaboration. • Use a trauma-informed approach to create resources and practices that promote MAiD provider resilience and engagement (including mindfulness, peer support, psychological support and case debriefing). • Practice continuous quality improvement through data tracking and engaging multiple stakeholders in the evaluation of our practice (families, clinical teams, leaders, ADRAS members, etc.).
**Team Structure**:
• Team members: ADRAS members from diverse professions and specialties provide 7–15 h/month to participate in MAiD practices (assessment, provision, team meetings, professional development). • Leadership: Operational Director and Physician Lead facilitate operational and clinical functions and team culture. • Care Coordinators: provide case coordination of MAiD referrals from first contact to follow-up; facilitate communication with other care teams; educate patients/families/clinicians about MAiD; ensure compliance with standards; promote continuous quality improvement (see Simpson-Tirone et al., 2022).

## Methods

### Program Evaluation Purpose and Rationale

Since August 2016 the ADRAS team has received three to six requests for MAiD per week and provides assisted deaths for HHS patients both in hospital and in the community. The purpose of this program evaluation study was to examine the effectiveness of the interprofessional model of care, and the policies, education and infrastructure that were put in place to support MAiD at HHS. Specifically, the study aimed to ascertain the perceptions of frontline HCPs (healthcare professionals and physicians), hospital leaders, and MAiD-involved families^1^, regarding the following questions:

1. What are the perceptions of the MAiD program amongst stakeholders, two years after its introduction?
2. Has the HHS MAiD program delivered on its intention to create a clinical service that aligns with the values identified in the stakeholder consultation/needs assessment process?
3. How can the HHS MAiD program improve on its mandate and care model?
4. What are the key components in creating and sustaining a program that delivers high-quality, whole-person MAiD care that could be translated to other organizational settings?

### Study Design

The evaluation was done using a mixed-methods research design, which included an online survey with Likert scale and open-ended questions, as well as focus groups and interviews with staff and physicians at HHS (Hsieh &Shannon, 2005). We also conducted interviews with senior leaders. Family members were interviewed 15–36 weeks after the death of their loved one. We used a mixed-methods design because it is the best way to describe social data in complex environments, including understanding values, beliefs and concerns of heterogeneous populations through a process called triangulation (Thurmond, 2001; Tracy, 2010). Survey and focus group questions were developed around the espoused values and goals of the MAiD program described in Fig. 1 (see Appendices 1, 2, and 3 for the survey, focus group and interview questions). For details on data collection and analysis procedures please refer to Appendix 4. The study was reviewed and approved by the Hamilton Integrated Research Ethics Board.

All HCPs, leaders and physicians working in adult care programs during the study period were invited to participate in the survey (approximately 3000 people in total). HCPs working in programs where MAiD requests had occurred (oncology, internal medicine and palliative care) were invited to participate in focus group discussions about their experiences with the MAiD program.

## Results

### Survey Sample

The online survey was completed by 356 health care professionals. Data from 56 respondents were excluded from analysis due to missing data; the threshold for inclusion in data analysis was surveys with ≥ 50% questions answered. The final sample for analysis was 300. Survey participants were primarily female (79%) and over half (55%) had been working in their professional role for fifteen years or more.

Front-line clinical staff comprised 87% of the respondents. The survey sample included individuals from a wide variety of professions (see Table 2). The majority of survey respondents (95%) came from the four inpatient adult acute care sites where the majority of MAiD requests had originated.

**Table 2:** Professions of Survey Respondents

Group N = 300 n (%)	Role/Specialty	N = 300 n (%)
Health Professionals 191 (63.6)	Nurses Social Workers Rehabilitation Science Workers Pharmacists Physiotherapists Respiratory Therapists Pharmacy technicians Allied Health Staff Speech Language Pathologists Occupational Therapists Dietitian Genetic Counsellors Radiation Therapists Recreation Therapists Behavior Therapists Home Care Worker	105 (35.0) 20 (6.7) 14 (4.7) 8 (2.6) 7 (2.3) 7 (2.3) 6 (2.0) 5 (1.7) 5 (1.7) 4 (1.3) 2 (0.7) 2 (0.7) 2 (0.7) 2 (0.7) 1 (0.3) 1 (0.3)
Physicians 70 (23)	Internal Medicine Palliative care Oncology Family Medicine Critical Care Anesthesia Pediatrics Laboratory Medicine Obstetrics Surgery Emergency Medicine Geriatrics Gynecology	24 (8.0) 11 (3.6) 10 (3.3) 8 (2.6) 7 (2.3) 5 (1.7) 5 (1.7) 2 (0.6) 2 (0.6) 2 (0.6) 1 (0.3) 1 (0.3) 1 (0.3)
Leaders 52 (17.3)	Administrative leaders Educators Unit Leaders	32 (10.7) 14 (4.7) 6 (2.0)
Support Staff 15 (5)	Support staff	15(5)

*Please note that the percentages of different professions reported exceed 100% because some individuals reported more than one profession

### Focus Group Sample

A total of 39 participants attended 6 different focus groups (Table 3).

**Table 3:** Characteristics of Focus Group Participants

Variable	Participants N = 39 n (%)
Gender Female Male	30 (76.9) 9 (23.0)
Age Range 18–24 years 25–34 years 35–49 years 50–64 years 65 + years	1 (2.5) 7 (17.9) 17 (43.5) 11 (28.2) 3 (7.7)
Years in Practice 1–4 5–9 10–14 15–20 20+	5 (12.8) 5 (12.8) 10 (25.6) 5 (12.8) 14 (35.8)
Professional Role Physician Nurse Rehabilitation Science Professional Social Work OTA/PTA Pharmacist Spiritual Care/Chaplain Manager/Director Nurse Practitioner Dietitian	14 (35.8) 10 (25.6) 5 (12.8) 3 (7.7) 2 (5.1) 1 (2.6) 1 (2.6) 1 (2.6) 1 (2.6) 1 (2.6)
Physician Specialty (n = 14) Internal Medicine Palliative Care Family Practice Oncology	2 (5.1) 2 (5.1) 5 (12.8) 5 (12.8)

### Family Interview Sample

We approached family members connected to sixteen MAiD patients to participate in the study. Two of the family members we contacted for an interview refused, one due their grieving experience. Another individual, who shared positive things about the ADRAS team during the recruitment phone call, decided not to participate because they felt they were moving on in a positive way from the experience. In two instances, two individuals took part in a single interview, resulting in a total of sixteen individuals taking part in fourteen interviews about fourteen MAiD patients. All family members who participated in an interview were present for their loved one’s MAiD procedure. Interviews were conducted an average of 31 weeks after the patient’s death. (Table 4).

**Table 4:** Characteristics of Interview Participants

Relationship of Interview Participant to Patient who Received MAiD	Time Between MAiD Provision and Interview (in weeks)	Location of Provision
Son	31	Hospital
2 Male Friends	15	Hospital
Husband	40	Hospital
Son	36	Hospital
Daughter	46	Hospital
Wife	19	Hospital
Son	24	Hospital
Daughter	28	Home
Wife	32	Hospital
Sister	32	Home
Son	31	Hospital
Daughter	27	Hospital
Wife & Daughter	37	Home
Daughter	40	Hospital

### Senior Leader Interview Sample

We interviewed three of the hospital corporation’s executive leadership team, and one board member familiar with the MAiD program.

## Findings

Findings across all data sets focused on: (1) perceptions of quality of MAiD care; (2) effectiveness of MAiD education and resources. In the discussion, we describe the relationship between the data collected in this program evaluation, and the needs and concerns identified in the original organizational ethics engagement project, to determine how successful the MAiD program designed on the basis of this earlier project was in meeting the needs of stakeholders.

### MAiD Family Perceptions of Quality of Care

The majority of family interview participants expressed high satisfaction with the quality of MAiD care their loved one received, commenting on the caliber of the ADRAS team members in terms of their compassion, professionalism and expertise, and the support they provided which created a high quality, therapeutic relationship.

*I don’t think in the process that we as a family experienced, I don’t think that it could have been better, given the constraints of the current legislation. I don’t think that anything could have been better. It was extremely well done with a massive amount of empathy and understanding and sensitivity*. (Wife, ID 009)

*They [members of the ADRAS team] were so absolutely on top of the game and so nice and so, as I said, solicitous without…without being obsequious. And they would answer any question that you had. They were…in that sense, magic in terms of the job that they were doing and they did it effortlessly, seamlessly. There was no angst or anything of that nature in the room and that was…it was absolutely fantastic*. (Friend ID 002)

The majority of family members described the MAiD provision in positive terms using words such as peaceful, dignified, and respectful.

*They provided anything that we wanted. They allowed family members that my mom wanted there. They allowed whoever she wanted there. There was never, “No, you can’t do that. You can’t do that. You can’t do that.” It was never, never a concern. Yeah, it was good. It was nice. It was dignified and peaceful and respectful and…like everybody should go.”* (Son, ID 001)

Some family members identified quality issues of a procedural nature, including: exasperation when the ADRAS team showed up a few minutes late; anger at the legally mandatory ten day reflection period; and frustration at the requirement that two independent witnesses needed to sign the patient’s MAiD request form (as required by the Canadian MAiD legislative framework at that time). These quality issues were not related to the performance of the ADRAS team per se, and some could not be rectified as they are required by federal law. However, frustration with finding available witnesses did prompt the MAiD program to identify a group of on-site HCPs willing to be independent witnesses for patient MAiD requests. On the whole, however, family members reported high satisfaction with the quality of care provided to them and their loved ones by the ADRAS team throughout the MAiD process (see also Frolic et al., 2020).

### Health Care Professionals’ Attitudes Toward the MAiD Program at HHS

#### Approach to Analysis

Survey data were grouped by type of survey participants’ involvement with MAiD over the previous two years: direct involvement with the MAiD process, education experience with the MAiD team, and no involvement with the MAiD program. This was done to understand the extent to which their perceptions of the effectiveness of the program in meeting its goals were based on first-hand experiences with ADRAS, or based instead on the reputation of the team.

Approximately one third of participants fell into each of these three categories, as detailed in Table 5. Since the distribution was so close to equal across the three categories, and since the number of respondents that fell into each category was so close to 100, we report percentages only in Tables 6 and 7.

**Table 5:** Survey Participants’ Level of Involvement with the MAiD Program at HHS

Direct Involvement with the MAiD Process N = 100 (33.33%)	Education Experience with the MAiD Team N = 105 (35%)	No Involvement with the MAiD Program N = 95 (32%)
I have had general conversations with patients and families about MAiD as an option.	I have attended an education session(s).	No involvement due to clinical need.
I have been involved in responding to a patient’s specific request as a member of the care team.	I have accessed the material on the HHS MAiD intranet site.	No involvement due to conscientious objection.
I have been directly involved in a MAiD assessment.	I have accessed the ADRAS team for coaching and support.	
I have been directly involved in a MAiD provision.		
Involved with family or friends who have requested MAiD.		

Results from the open-ended survey questions and focus groups were organized into the three main themes that characterize the pillars of high-quality MAiD care as expressed in the stakeholder needs assessment project conducted at HHS in 2015: (1) Honoring Diversity, (2) Supporting Values, (3) Enabling Resources (see Fig. 1). The rationale for this approach to analysis was to evaluate how successful the service had been in meeting the identified needs of HCPs and aligning practice with the espoused values of the MAiD program in its first two years of operation.

#### Health Care Professionals’ Confidence in the MAiD Program at HHS

Data describing health care professionals’ attitudes towards the MAiD program at HHS were organized into two categories: health care professionals’ confidence in the MAiD program at HHS; and the perceived effectiveness of MAiD education and resources.

The level of agreement with the survey statements about respondents’ confidence in the MAiD program at HHS is related to their level of experience with the program (Table 6). Over three-quarters (and often over 80%) of individuals who had direct experience with the program agreed with the statements about confidence in the program. Over two-thirds of those with education experience agreed with the confidence statements.

However, having no experience with the MAiD program does not equate to negative perceptions about the program. For individuals with no experience with the MAiD program, the neutral rating is higher than the disagree rating.

**Table 6:** Health Care Professionals’ Confidence in the MAiD Program at HHS by Level of Experience with the MAiD program

	Direct Involvement with the MAiD Process % (N = 100)			Education Experience with the MAiD Team % (N = 105)			No Experience with the MAiD Program % (N = 95)		
	Agree	Neutral	Disagree	Agree	Neutral	Disagree	Agree	Neutral	Disagree
I am confident that the ADRAS team can provide appropriate education/support for all healthcare professionals responding to requests for MAiD.	78	20	2	72	23	5	54	35	11
I am confident that patients requesting MAiD will have access to a range of end of life options including palliative care, to support informed choice.	84	11	5	75	16	9	58	31.5	10.5
I am confident the ADRAS team can deliver high quality assessment and care for patients who request MAiD.	81	16	3	73	22	5	49.5	41	9.5
I feel HHS has created a culture of respect for moral diversity re: MAiD (supporting healthcare professionals’ choice to participate or not participate).	88	6	4	69	22	9	43	47	10
I am confident the privacy of healthcare providers and patients/families participating in MAiD will be protected appropriately, to the degree they wish.	86	13	1	81	13	6	59	36	5
I feel HHS supports patient autonomy in its design of MAiD services.	84	12	4	73	25	2	47	47	6

#### The Effectiveness of MAiD Education and Resources

The level of agreement with the survey statements about MAiD education and resources at HHS was also related to level of experience with the program (Table 7). This indicates the messaging and education strategies have been effective in facilitating an understanding of the practice.

Survey respondents who report having no experience with the MAiD program shift towards neutrality, not disagreement in their ratings of the survey statements, which indicates that by reputation the education dimension of the MAiD program was strong, as was the appetite for more education.

**Table 7:** Health Care Professionals’ Perceptions about MAiD Education and Resources at HHS by Level of Experience with the MAiD program

	Direct Experience with MAiD Process % (N = 100)			Education Experience with the MAiD Team % (N = 105)			No Experience with the MAiD Program % (N = 95)		
	Agree	Neutral	Disagree	Agree	Neutral	Disagree	Agree	Neutral	Disagree
I know how to access information for patients/families if they ask about the option of MAiD.	79	14	7	68	20	12	32	24	44
The resources and tools developed by HHS support a timely, consistent response to patient requests for assisted dying.	71	24	5	60	36	4	22	67	11
I understand my professional responsibilities for responding to patient requests for MAiD	81	15	4	68	17	15	35	38	27
I feel I’ve received adequate opportunities to learn about MAiD and its practice at HHS.	64	25	11	54	22	24	25	27	47
I understand the role of the Assisted Dying Resource and Assessment Service (ADRAS).	79	10	11	65	20	15	28.5	29.5	42

One respondent explicitly linked a change in their views on MAiD to the ADRAS team:

*I feel that the resources and education provided have contributed greatly to advancing my comfort level as well as staff’s comfort level in being able to respond to patients and families should they ask questions. I also believe that staff and I feel more empowered to speak to treating physicians should their patient request MAiD. Our teams now bring the discussion to team rounds as a standard of practice when there is an inkling that a patient or family may want to discuss this.*

Overall, based on the survey, within the first two years the ADRAS team was highly effective in meetings its goals to provide safe, effective, whole-person and values-based MAiD care to patients and their families, as well as education and supports for HCPs. Positive attitudes towards the MAiD program were highest amongst those with direct experience with MAiD, but were also strong in those with no clinical or educational exposure to the ADRAS team. This means that the ADRAS team had a strong reputation for providing high quality care across the hospital, and that it had been effective in meeting its mandate to provide education and capacity-building opportunities.

## Pillars of High Quality MAiD Care

### First Pillar of High-Quality MAiD Care: Honoring Diversity

The majority of participants in the 2015-16 stakeholder needs assessment project identified the need to ensure the values of all staff are respected, including values around conscientious objection to MAiD or participation in MAiD (Frolic et al., 2022b).

Two-thirds (66%) of survey respondents supported the statement: “I feel HHS has cultivated a culture of respect for moral diversity re: MAiD.” Just over a quarter of respondents (27%) responded “neutral” to this statement and only 7% disagreed with this statement; indicating that respecting moral diversity was identified as having been done well in the process of developing MAiD resources, services and practices at HHS.

However, in both the survey and focus group data some participants described their perception that support for MAiD has increased and conscientious objectors as a minority may not always feel comfortable sharing their views. A palliative care nurse explained:

*I think the tables have turned and those of us who are conscientious objectors are shrinking and some people, who maybe were on the fence, are coming around to being comfortable with it or at least feeling, you know, better about it than they did before it actually happened. And I just feel like it’s unveiled in the room sometimes that, you know, nobody wants to identify as a conscientious objector anymore as our numbers seem to be small, or at least that’s the perception, because people aren’t talking about it.*

Similarly, an oncologist described how there was a fairly sudden change in the culture around MAiD and how this shift may be causing some people to lose sight of the fact that not everybody is comfortable with the MAiD:

*I’ve sat in meetings with Directors where the prevailing sort of discussion is that MAiD is just the norm. And I don’t think that that’s necessarily the case … I think MAiD is a requirement but it’s not the norm. There wasn’t even the thought process that I might think differently … So, that’s why I think that there’s been a culture sort of change and it flipped … it didn’t happen gradually*.

The need for support for conscientious objectors was identified as a concern by some respondents in the open-ended survey data. However, those who articulated a strong objection to MAiD vocalized opposition to the legal decriminalization of MAiD, and the College of Physicians and Surgeons of Ontario’s requirement that physicians must make an effective referral for patients seeking MAiD. Thus, their primary objections were legal and professional in nature, rather than specific to the MAiD program at HHS.

Thus, in general, the data indicate that staff and physicians believe HHS has been successful in creating a culture of respect for moral diversity on the issue of MAiD. However, the rapid uptake of MAiD over the past two years has caused some conscientious objectors to feel somewhat marginalized, and suggests that some additional supports for conscientious objectors may be needed.

### Second Pillar for High Quality MAiD Care: Supporting Values

To identify how successful the MAiD program had been in meeting its goals, as well as gaps in the achievement of its aspirations, we mapped program evaluation findings onto the following key values identified by participants in the stakeholder consultation in 2015-16:

- Autonomy: Respect for patients’ right to choose MAiD as an option.
- Privacy: Protecting the privacy and confidentiality of patients and providers of MAiD.
- Beneficence: Providing safe and high-quality MAiD care for patients and families.

#### Respect for patient autonomy and access

Two-thirds (66%) of all survey respondents supported the statement: “I feel HHS supports patient autonomy in its design of MAiD services”. Both the survey and qualitative data identified the leadership of HHS in providing patients with access to high-quality MAiD care through the ADRAS team; however, consistent and timely access to MAiD was identified as a significant concern in the open-ended survey data (Table 8).

**Table 8:** Concerns Related to Patient Access to MAiD

Concerns Related to Patient Access to MAiD
• Insufficient awareness amongst HCPs that MAiD is an option for both inpatients and outpatients in community
• MAiD is not presented as an option for patients unless they ask about it
• Some HCPs will not hear or act upon patient questions about MAiD due to their own personal views on MAiD, creating a barrier to access
• No ability for patients to self-refer for a MAiD assessment
• Timeliness of ADRAS service response

One participant described her concerns about awareness of the MAiD program: “*It’s not being advertised (probably the wrong word but you get my point) enough. Do people even know it is an option? Probably not in my opinion*.” Some survey respondents explicitly identified concerns around “*how and when to access the service when the Most Responsible Physician is a conscientious objector*” and that “*some staff may bring their own feelings and prejudices and not listen to clients/families’ needs and requests*.” These comments indicate that there is a tension between respecting the moral diversity of staff/physicians and their right to object to MAiD, with the right of patients to access MAiD.

Timeliness of access to MAiD was raised as a concern by some survey respondents with a few respondents expressing concern about the time required for the assessment/approval process. Some noted that patients in the hospital are often acutely ill and deteriorate very quickly, potentially rendering them ineligible for MAiD as their capacity to consent to this procedure at the time of provision could wane with advancing illness.

Thus, while the majority of respondents indicate that HHS supports patient autonomy and access to MAiD, many raised concerns illustrating that in practice there are some logistical and social barriers that patients still encounter when attempting to access MAiD, including access to appropriate and timely information and referrals to this end of life care option.

#### Privacy: protecting privacy and confidentiality of patients and providers of MAiD

Nearly three-quarters (74%) of all survey respondents supported the statement: “I am confident the privacy of healthcare providers and patients/families participating in MAiD will be protected appropriately, to the degree they wish.” Qualitative data also indicated that in general, staff and physicians felt confident in the procedures put in place to protect the confidentiality of patients receiving MAiD.

However, the privacy of patients and families was identified by some respondents as a concern in the open-ended survey data. A few respondents suggested the need for a private space for MAiD provision, “*As this is such a private family matter, it would be good to have a dedicated room at each site for MAiD, for the family’s comfort and privacy*.”

Focus group data identified the need for a careful balancing of values, between protecting the patient’s privacy and also ensuring that clinical staff are aware of the patient’s request for MAiD, so that appropriate supports can be put into place to support both the patient/family and clinical staff caring for the patient around the time of a MAiD procedure.

#### Beneficence: providing safe and high-quality MAiD care for patients and families

Across all survey respondents, (68%) agreed with the statement about the ADRAS team delivering high-quality assessment and care for patients who request MAiD. Patient care was identified as one of the things that had been done well in the development of the MAiD program at HHS. Participants described that care was “*holistic*” and “*patient-centred*” and that “*health care providers are very kind to the patient*” in the context of MAiD.

One respondent described:

*My experience with MAiD was very positive and the family and patient also provided positive feedback re: the process. It really helped the family to have the consistent support of the ADRAS team as well as the unit team before, during and after the provision*.

Concerns were identified in the open-ended survey data around providing support for patients who weren’t eligible to receive MAiD, and for providing support for families before, during and after provision, in collaboration with clinical teams.

Additional resources for supporting patients and families included: specialized counselling services for families during the MAiD process, bereavement sessions or support groups for families after MAiD provision, and support for ineligible patients.

### Third Pillar of High-Quality MAiD Care: Enabling Resources and Infrastructure

Evaluation data collected related to the infrastructure of the MAiD program were organized into three themes: education and capacity building; the ADRAS team; and sustainability of the program.

#### Education and Capacity Building

In the survey data, over half (58%) of respondents indicated that they know how to access information for patients/families about the option of MAID. Nearly two-thirds (61%) indicated they understand their professional responsibilities for responding to patient requests for MAiD. Open-ended data indicate that many survey respondents believed education was something that had been done well in the development of the MAiD program, with some respondents referring to “*great education resources on the intranet*”, and others referring to “*excellent education sessions*”. Some survey responses recognized the role of the ADRAS team in facilitating education: “*The ADRAS team has been a great resource for meeting the gaps in frontline staff knowledge of MAiD*”.

However, education and capacity building were also identified as opportunities for future improvement as just under half (48%) of survey respondents supported the statement, “*I feel I’ve received adequate opportunities to learn about MAiD and its practice at HHS.*” Many respondents identified the need for ongoing education for HHS staff, including periodic updates. In the open-ended survey questions, staff also suggested making a website for patients/families who have questions about MAiD.

The focus group data emphasized the need to develop clinician capacity in exploring patients’ end of life wishes and hearing and responding to requests for MAiD. A nurse practitioner suggested:

*If there was almost more sensitive scripting for the MAiD program exploring people’s wishes that way, I would probably use it to sort of tease out, are they just saying that or are they really serious? … I actually have someone, they’ve told me that they just want to die, and I’ve been trying to think about how do I approach this?...even just a small scripting guide would be helpful*.

In summary, both the survey and qualitative data highlight the general success of the MAiD program’s investment in education, resource-development and capacity-building across the organization to promote awareness of the resources and structures to support patients, families and clinicians through the MAiD process.

#### Collaboration with Palliative Care

In the stakeholder needs assessment project, palliative care physicians strongly identified as conscientious objectors, and expressed skepticism about the possibility of providing truly values-based, whole-person MAiD care. However, in the program evaluation two years later, palliative care physicians’ perceptions about the MAiD services were quite positive, with many describing satisfaction with the resources developed, with MAiD patient access to palliative care resources, and the respect demonstrated by the ADRAS team. Even the challenges identified (such as concern that the ADRAS team may not have the competence to explore with patients other end of life treatment options) were largely framed as opportunities for deeper collaboration between palliative care and MAiD practitioners, rather than concerns about the quality of MAiD services.

#### The MAiD Team (ADRAS)

In our mixed methods study, every data set revealed a strong theme related to the strength of the interprofessional team model of the ADRAS team for its delivery of high-quality, personalized and sensitive care to patients, in collaboration with the clinical teams surrounding the patients.

Just half (58%) of the survey sample indicated they understand the role of the Assisted Dying Resource and Assessment Service (ADRAS), with 22% indicating they didn’t understand the role of ADRAS and 20% responding neutral to this item.

Nevertheless, over two-thirds (68%) felt confident that the ADRAS team can: provide appropriate education/support for healthcare professionals responding to requests for MAiD, and deliver high quality assessment and care to patients who request MAiD. Only 6% didn’t feel confident about these statements and just over a quarter (26%) responded neutral to these items.

It is interesting that confidence in the team is reported as higher than understanding of the role of the team. This speaks to the reputation of the team as a provider of high-quality care, even amongst those without direct exposure or total understanding of the team’s role.

Many respondents expressed gratitude to the ADRAS team, for example:

*Thank you for your support. Our staff values the accessibility and approachability of the ADRAS team. Your willingness to come to them to meet their educational needs in a timely manner has truly been an asset*.

Concerns identified about the ADRAS team through the open-ended survey data include: access to independent assessors (apart from the ADRAS team), and the need for enhanced collaboration with clinical programs in care planning for patients.

#### Sustainability

While the program evaluation study confirmed that the MAiD program at HHS had achieved its goals of creating a service that honors diversity, supports values and has enabling resources and standards to build capacity and facilitate access to high quality MAiD care, a new theme of sustainability was raised in the evaluation data. The qualitative data specific to sustainability fell into two main categories: financial sustainability and resilience/human sustainability. Financial sustainability refers to the costs (direct and indirect), resources and financial impacts of operating the ADRAS team. Resilience/human sustainability refers to the human costs (psychological and otherwise), associated with being a part of MAiD practice.

#### Infrastructural sustainability

In interviews with senior leaders, participants articulated concern about the perpetual budget pressures within the hospital and the risk this poses to a small clinical service like ADRAS. Specifically, leaders recognized that case coordination can be very time consuming, and having a designated care coordinator is essential to the team’s function and to ensuring patient inquiries are responded to in a timely manner. As well, the team’s medical and operational leadership were perceived as foundational; their roles in managing the social and administrative dimensions of the program help to liberate ADRAS members to focus their efforts and energy on the patients and their families to deliver the best possible care.

#### Resilience and human resource sustainability

Leaders expressed pride and confidence in the innovation and capacity of ADRAS. However, they also identified the increased risk of burn out, compassion fatigue and distress associated with high-risk practices like MAiD, and the importance of creating safe team practices and spaces to ensure the psychological wellbeing of MAiD providers. Limiting the caseload of individual ADRAS team members to ensure adequate time between cases, was identified as an important resilience strategy to keep engagement high and avoid exhaustion. Leaders recognized that prioritizing sustainability may mean occasionally triaging cases or referring patients elsewhere when experiencing a large volume of referrals.

## Discussion

This mixed methods program evaluation was successful in gathering data from a wide range of stakeholders—including family members, health care professionals and senior leaders—to determine the successes of the MAiD program at HHS, to identify opportunities for quality improvement and to provide direction to support the sustainability of the program into the future. It enabled a comprehensive evaluation of the MAiD program at HHS in the context of the three pillars to support high-quality care identified during the readiness assessment: Honoring Diversity, Supporting Values and Enabling Resources, as summarized in Fig. 1.

Strengths of this evaluation study include: our large sample of health care providers; the diversity of disciplines and stakeholders represented; and the spectrum of opinion represented in the sample. Both survey and focus group participants included individuals who identify as conscientious objectors and individuals who identify as supporters of MAiD. Family of MAiD patients who interacted with a wide variety of ADRAS members provided feedback on their care experience. Through our evaluation we captured the lived experience (direct and indirect) of health care professionals working in an organization practicing MAiD. Our program evaluation focused on the concrete espoused values of the MAiD program rather than general satisfaction questions.

Limintations of our study include: was conducted at a single center, in a large, academic, tertiary care hospital which may limit the generalizability of our results. Focus group members were self-selected and thus may not represent the range of HCP experiences. Another limitation of our study is that it does not report on patient experience with the MAiD program. Our findings are similar to other studies in relation to the complexities experienced by MAiD families in other Canadian studies, but differs significantly in its methods of data collection. Our family interviews were conducted by a non-clinical member of the research team, and occurred in non-clinical locations where the participant did not experience their MAiD loss. The intention was to create a supportive, trauma-informed environment, facilitating a comfortable interview where participants had the space to speak honestly and candidly, and minimizing the risk of harm to participants.

Our program evaluation study indicates the following quality indicators may be relevant for other hospital-based MAiD programs:

- Respect for moral diversity: support is provided for conscientious participants and objectors alike.
- Timely access: standardized referral processes and care coordination ensure patient inquiries are responded to rapidly and compassionately.
- Whole person care for patients and families: MAiD providers attend to the psycho-emotional-social needs of patients and families, not just the technical dimensions of care.
- Education and capacity-building: all stakeholders need access to relevant and accessible educational resources about the legal and clinical aspects of MAiD; a MAiD program has a critical role in developing the capacity of clinical teams to support patients and families through the process.
- Resilience and sustainability: sustainability of a MAiD program requires ongoing recruitment of new members to ensure adequate human resources are available to meet increasing demand and avoid burnout; the development of resilience practices and resources and psychological safety within the MAiD team; and engagement of senior leader support.

Areas of future research include: the development of a formal set of quality indicators (i.e. a program “dashboard”) and the collection of quantitative metrics to support ongoing program accountability; as well as checkpoints to understand how perceptions of quality change over time, especially when eligibility criteria and safeguards change as MAiD legislation is revised or expanded to include new populations.

## Conclusions

We conducted this program evaluation two years after MAiD became accessible in Canada, at a time when MAiD was still considered to be a novel practice. This study was motivated by our ethical imperative to evaluate this new clinical service in order to test the fidelity of the program against the needs identified by stakeholders and the values that informed the design of the ADRAS team. We wanted to understand whether the ethical standards espoused by the MAiD program were being lived out in practice. Early and rigorous program evaluation allowed us to identify gaps in quality, to take the pulse on the reputation of this new, controversial program, and to address quality issues proactively in order to support the sustainability of the program and continuously enhance service to all stakeholders. Most importantly, this study enabled us to overcome biases in our perceptions of the quality of MAiD services by hearing how diverse participants experience the MAiD program.

As MAiD becomes more common in jurisdictions around the world, and becomes subject to increasing public and regulatory scrutiny, healthcare organizations may consider using a mixed methods program evaluation approach like this one to: understand the reputation of the program; to identify its strengths and impacts; to mitigate risks and challenges; to ensure adherence to ethical standards; and to amplify the trust of all stakeholders in the espoused values governing MAiD practice.

## Data Availability

The consent form signed by participants in our study did not include consent for sharing data beyond what is reported in this manuscript which means that data are not available for sharing.

## Appendix 1 – Survey

***1. What is your gender?***

Female, Male, Prefer not to disclose, Gender not listed (specify).

***2. What is your age range?***

18–24, 25–34, 35–49, 50–64, 65+.

***3. How many years have you been in your practice?***

1–4, 5–9, 10–14, 15–20, 20+.

***4. What is your primary professional role? (Check all that apply)***

Physician, Nurse, Spiritual Care/Chaplain, Social Work/Counsellor, Psychotherapist, Pharmacist, Rehab Science, Home Care/Support Worker, Other (please specify).

***5. If you selected physician, which medical specialty describes your practice?***

General Practitioner, Oncologist, Palliative Care, Geriatrics or Elder Care Specialist, Anesthesiologist, Internal Medicine, Neurologist, Psychiatrist, Paediatrician, other (please specify).

***6. Which HHS facility do your work at? (Check all that apply)***

Juravinski Hospital and Cancer Center, Hamilton General, West Lincoln Memorial Hospital, St. Peter’s Hospital, MUMC, Other.

***7. What has been your involvement in MAiD to date? (Check all that apply)***

* I have attended an education session(s) on MAiD at HHS; * I have reviewed the material on the HHS MAiD intranet site (http://corpweb.hhsc.ca/body.cfm?id=4435); * I have educated myself about MAiD through other sources (i.e. information from my college/professional organization, news articles, etc.); * I have had general conversations with patients and families about MAiD as a care option; * I have been involved in responding to a patient’s specific request for MAiD as a member of the care team; * I have accessed the ADRAS team for coaching and support; * I have been directly involved in MAiD assessment or provision.

Other (please specify).

**Likert Scale Questions**: (Strongly Agree, Agree, Neutral, Disagree, Strongly Disagree)

**8.** I know how to access information about MAiD if patients/families ask about the option of MAID.

**9.** The resources, tools and structures developed by HHS help to support a timely, consistent response to patient requests for assisted dying.

**10.** I understand my professional responsibilities for responding to patient requests for MAiD.

**11.** I feel I’ve received adequate opportunities to learn about MAiD and its practice at HHS.

**12.** I understand the role of the Assisted Dying Resource and Assessment Service (ADRAS).

**13.** I am confident the ADRAS team can deliver high-quality assessment and care to patients who request MAiD.

**14.** I am confident that the ADRAS team can provide appropriate education/support for healthcare professionals responding to requests for MAiD.

**15.** I am confident that patients requesting MAiD will be supported to access a range of end of life care options, including palliative care, to support informed choice.

**16.** I feel HHS supports patient autonomy in its design of MAiD services.

**17.** I am confident the privacy of healthcare providers and patients/families participating in MAiD will be protected appropriately, to the degree they wish.

**18.** I feel HHS has cultivated a culture of respect for moral diversity re: MAiD (i.e. supporting healthcare professionals’ choices to participate or not participate in MAiD).

**Short Answer Questions**:

**19.** What do you think has been done well in the process of developing MAiD services and practice at HHS? 20. What are your primary concerns/worries about MAiD services and practice at HHS?

**21.** Do you feel you require additional resources/supports to respond to MAID questions by your patients or families? If yes, please provide examples.

**22.** Has your view of MAiD changed over the last year? If so, how?

**23.** What opportunities do you see for improvement to or further development of MAiD services and practices at HHS ?

**24.** Is there anything you wish to add?

## Appendix 2: Detailed Methodology for the MAiD Program Evaluation

**Online Survey Data Collection Methods**.

The survey was constructed using Survey Monkey software and included the following groups of questions:

- Questions that explored the demographics of the respondents.
- Likert scale questions (using a 5-point Likert scale ranked from Strongly Disagree to Strongly Agree) exploring staff/physician perceptions of various aspects of the MAiD program.
- Open-ended text questions that addressed concerns, values and potential for improvement of the MAiD program.

Survey Recruitment Methods

An email stating the purpose and importance of completing the online survey along with a consent statement and a link to the online survey was distributed through current email lists to physicians, healthcare professionals, and leaders at Hamilton Health Sciences. The survey was also advertised in staff newsletters and on the staff website.

Survey Fielding

The survey was launched on October 28/2017 and closed on January 02/2018. A reminder email was sent out the first week of December, following the same procedures described above in the recruitment methods section.

**Focus Group Data Collection Methods**

Focus Group Participant Recruitment

Recruitment for focus groups targeted clinical programs with the most MAiD requests at HHS over the past two years: oncology, palliative care, and medicine. An email invitation was sent to clinicians working in these three programs. Separate focus groups were held with physicians and interprofessional staff working in these programs because their roles and responsibilities related to the components of MAiD practice (referral, assessment and provision) are different. Interprofessional staff included: social work, nursing, clinical managers, spiritual care, rehabilitation, speech language pathology, and pharmacy.

The email invitation described the purpose of the focus groups in the context of the MAiD program evaluation and explicitly stated that individuals with a variety of perspectives and experiences related to MAiD were welcome to attend, not just those who have participated directly in MAiD.

Focus Group Data Collection

We conducted six focus groups with HHS clinicians from programs representing patient populations most frequently requesting MAiD: oncology, palliative care, and medicine.

The focus groups were approximately 60 min in length and a light meal was provided. Participants were asked to complete a short demographic form prior to the discussion. The facilitator used a semi-structured focus group guide to generate discussion and reflections from participants about their experiences with, and perceptions about the MAiD program. The focus groups were digitally recorded and the recordings were transcribed verbatim by a professional transcriptionist. The transcripts were anonymized prior to analysis.

**Senior Leaders Data Collection Methods**

We conducted interviews with the Directors of the MAiD program as well as four senior leaders at HHS responsible for oversight of the MAiD program, as well as a hospital board member. Interviews were scheduled at a time and location that was convenient for them and were 30–40 min in length. The interviewer used the semi-structured interview guide to generate discussion and reflection from the senior leaders about their experiences with, and perceptions about the program. The interviews were digitally recorded and transcribed verbatim by a professional transcriptionist. The transcripts were de-identified and anonymized prior to analysis.

**Family Member Interviews Data Collection Methods**

During 2018–2019 we conducted semi-structured interviews with family members who had a loved one receive MAiD through ADRAS. Family members were purposefully sampled to include patients who received MAiD at three different hospital sites as well as in the patient’s home, and from different providers on the ADRAS team.

Family Interviews Recruitment Process

We phoned family members who had given permission to be contacted for research about the MAiD program during their bereavement phone call. During this initial phone call, we described the purpose of the interview and asked if they were willing to participate in an interview.

Family Interviews Data Collection

Interviews were scheduled at a time and place that was convenient for the family member, either in person at the McMaster University Medical Centre (a neutral location as no MAiD services are provided there) or by phone as per the family member’s preference. One family member was interviewed in their home at their request. Refreshments and parking passes were provided for family members who were interviewed at the McMaster University Medical Centre.

**Survey Data Analysis Procedures**

Quantitative data were tabulated anonymously using the Survey Monkey software. Data from the open-ended questions were analyzed using conventional content analysis to identify themes. Two members of the research team (MS and LM) reviewed the data from the open-ended questions and highlighted words in the text that captured key thoughts or concepts. Labels for codes were developed based on the highlighted words in the data and a preliminary list of codes was developed. All open-ended data were independently coded by two team members (MS and LM) and coding was finalized through consensus at a coding meeting. When the coding was completed, the research team reviewed coding reports and then met to organize the codes into meaningful categories based on their relationships to each other. The qualitative software package NVivo (Version 11.0) was used to manage the open-ended survey data.

**Qualitative Data Analysis Procedures**

The transcripts from the focus groups and interviews were analyzed using conventional content analysis (Hsieh & Shannon, 2005) to identify themes through a qualitative descriptive approach (Sandelowski, 2000).

In our study, three members of the research team (MS, AO, LM) reviewed three transcripts (2 focus group transcripts and one interview transcript) in their entirety to understand the context of the discussion and then completed line by line coding of the transcripts, highlighting words in the text that capture key thoughts or concepts. These three individuals then met to discuss the codes and develop a preliminary list of codes through consensus. The analyst (MS) coded the remaining transcripts, noting any new codes in the study audit trail (Rodgers & Cowles, 1993) and ensuring new codes were applied to previously coded transcripts. The PI (AF) reviewed all transcripts to inform the development of the coding schema.

When coding was complete, research team members reviewed coding reports and then met to organize the codes into meaningful categories based on their relationships to each other and to ensure that there was an exemplar from the data for each code and category. The categories of codes were then organized into higher level clusters (Patton, 2002). The analyst recorded all decisions related to the coding and analysis process in a study audit trail (Rodgers & Cowles, 1993). The research team assessed data saturation through a review of all transcripts, coding reports and a review of the audit trail.

**Mixed Methods Analysis Procedures**

The findings from the survey were used to inform probes for the open-ended questions in the focus groups and interviews. Findings from the focus groups and interviews are be interpreted in the context of the survey findings with similarities and differences noted through a process called triangulation (Thurmond, 2001).

## Footnotes

1 Note: Family members” and “loved ones” are used interchangeably throughout the paper to refer to people of primary importance to the lives of patients, be they friends or relatives by birth or marriage. All family members/loved ones included in this study were asked by the patient to be present at their MAiD death.

## Declarations

### Registration

This study did not require registration.
